# Ivabradine Treatment Reduces Cardiomyocyte Apoptosis in a Murine Model of Chronic Viral Myocarditis

**DOI:** 10.3389/fphar.2018.00182

**Published:** 2018-03-05

**Authors:** Ge Li-Sha, Liu Li, Zhou De-Pu, Shi Zhe-Wei, Gu Xiaohong, Chen Guang-Yi, Li Jia, Lin Jia-Feng, Chu Maoping, Li Yue-Chun

**Affiliations:** ^1^Department of Pediatric Emergency, The Second Affiliated Hospital and Yuying Children’s Hospital of Wenzhou Medical University, Wenzhou, China; ^2^Department of Cardiology, The Second Affiliated Hospital and Yuying Children’s Hospital of Wenzhou Medical University, Wenzhou, China; ^3^Department of Cardiology, The First College of Clinical Medical Sciences, China Three Gorges University, Yichang, China; ^4^Children’s Heart Center and Department of Pediatrics, The Second Affiliated Hospital and Yuying Children’s Hospital of Wenzhou Medical University, Wenzhou, China

**Keywords:** coxsackievirus, chronic viral myocarditis, heart rate, ivabradine, cardiomyocyte apoptosis

## Abstract

This study was designed to explore the effects of ivabradine on cardiomyocyte apoptosis in a murine model of chronic viral myocarditis (CVMC). Mice were inoculated intraperitoneally with Coxsackievirus B3 at days 1, 14, and 28, respectively. On day 42, the mice were gavaged with ivabradine for 30 days until the 72nd day. The heart of infected mice was dilated and a large number of interstitial fibroblasts infiltrated into the myocardium on day 42. Compared with the untreated CVMC mice, mice treated with ivabradine showed a significant reduction in heart rate and less impairment of left ventricular function on day 72. The positive apoptosis of myocardial cells in the untreated CVMC group was significantly higher than that of the normal group and was significantly reduced after treatment with ivabradine. The expression levels of Bax and Caspase-3 in the untreated CVMC group were significantly higher than those of the normal group and were apparently reduced in the ivabradine-treated group versus the untreated CVMC group. Bcl-2 showed a high expression in the normal group and low expression in the untreated CVMC group, but its expression level in the ivabradine-treated group were higher than that of the untreated CVMC group. These results indicate that ivabradine could attenuate the expression of Caspase-3 by downregulation of Bax and upregulation of Bcl-2 to prevent the deterioration of cardiac function resulting from ventricular myocyte loss by cardiomyocyte apoptosis.

## Introduction

In 1965, Mattingly (Mattingly, 1965) first described dilated cardiomyopathy (DCM) as a myocardial disease without valve dysfunction. Cardiomyopathy research has reached its peak in the last half century as it has gained increasing attention worldwide. Many researchers considered that enterovirus, especially Coxsackievirus B (CVB), was the most common cause of DCM, although other pathogenic factors of cardiomyopathy, such as viruses, bacteria, medicines and poisons, and systemic diseases (Bowles et al., 1986; Yajima and Knowlton, 2009; Venteo et al., 2010), were also reported. In recent years, an upward trend in the incidence of viral myocarditis in adolescents has been noted (Cooper, 2009). Epidemiological data have shown that myocarditis is the primary cause of heart failure in adults less than 40 years of age (Yajima and Knowlton, 2009). The heart experiences three main stages after viral infection (Bowles et al., 1986; Feldman and McNamara, 2000; Yajima and Knowlton, 2009). (1) Acute myocarditis: the virus directly damage the myocardium, destroys the cellular structure, replicates in the cells, and triggers the body’s immune response. This stage involves non-specific immune responses. (2) Subacute myocarditis: This phase is characterized by infiltration of inflammatory cells, including natural killer cells and macrophages, and the production of antibodies. Because the myocardial antigen is similar to the viral antigen, an autoimmune reaction is elicited wherein the antibodies damage the normal myocardium; this promotes the further development of myocarditis. (3) Chronic myocarditis: Most of the viral population disappears after infection. The sustained autoimmune reaction and inflammatory response of the myocardium lead to myocardial remodeling that develops into the chronic viral myocarditis (CVMC) phase, potentially even into DCM and heart failure. Epidemiological data have shown that in 25–33% of patients, acute viral myocarditis will develop into CVMC (Bowles et al., 1986; Henke et al., 2003; Yajima and Knowlton, 2009). Many researchers have shown that myocardial apoptosis plays an important role in the genesis and development of CVMC, in addition to inflammatory factors, oxidative stress, collagen, etc. (Huber et al., 1999; Nakamura et al., 1999; Feldman and McNamara, 2000; Liu and Mason, 2001; Henke et al., 2003; Joo et al., 2003; Kyto et al., 2004; Guo et al., 2010; Yue-Chun et al., 2012).

Kerr et al. (Kerr et al., 1972) proposed the concept of apoptosis for the first time in 1972. Cells that undergo apoptosis show different morphological features such as cell shrinkage, increased cytoplasmic density, chromatin condensation, nuclear DNA degradation, intracellular membranes surrounding the DNA fragment, and formation of the apoptosis body. Although the mechanism of apoptosis induction is not fully clear, many researchers consider that it involves three main signaling pathways: the mitochondrial signaling pathway, the death receptor signaling pathway, and the endoplasmic reticulum signaling pathway (Kerr et al., 1972; Chinnaiyan et al., 1995; Colston et al., 1998; Vander Heiden and Thompson, 1999; Antonsson et al., 2001; Mito et al., 2011).

Ivabradine (IVA) can selectively inhibit the sinoatrial node I_f_ current to slow down the sinus rhythm without any effects on intra-atrial, atrioventricular, or intraventricular conduction times; myocardial contractility; and blood pressure (Mulder et al., 2004; Bohm et al., 2010; Ceconi et al., 2011; Parakh and Bhargava, 2011; Tardif et al., 2011; McMurray et al., 2012; Yue-Chun et al., 2012). The SHIFT studies have confirmed that IVA can significantly reduce the all-cause mortality and rehospitalization rate of patients with chronic heart failure, alleviate cardiac dilatation, and improve the left ventricular ejection fraction (LVEF) (Mulder et al., 2004; Bohm et al., 2010; Ceconi et al., 2011; Tardif et al., 2011). Therefore, IVA was recommended for heart failure patients by the European Society of Cardiology in 2012 (McMurray et al., 2012). Our previous study revealed that short-term treatment with IVA could moderately reduce cardiomyocyte apoptosis in acute viral myocarditis (Yue-Chun et al., 2012). Further, IVA significantly inhibited the development of CVMC that lead to DCM (Yue-Chun et al., 2016), but the effect of IVA on cardiomyocyte apoptosis of CVMC remains unknown. The present study was therefore designed to explore the effects of IVA on cardiomyocyte apoptosis and its possible mechanism of action in a murine model of CVMC repeatedly induced with CVB3.

## Materials and Methods

### Ethics Statement

The investigation conformed to the Guide for the Care and Use of Laboratory Animals published by the US National Institutes of Health (NIH Publication, 8th Edition, 2011), and all experiments were carried out in accordance with China Animal Welfare Legislation and were approved by the Wenzhou Medical University Committee on Ethics in the Care and Use of Laboratory Animals. All experimental animals were sacrificed with an overdose of pentobarbital (100 mg/kg, one dose intraperitoneally).

### CVMC Mice Model

Ninety-six-week-old specific pathogen-free (SPF) inbred male BALB/c mice obtained from the Shanghai Laboratory Animal Center China were divided into 6 groups: Normal control 1 group (*n* = 10), CVMC1 group (*n* = 20), Normal control 2 group (*n* = 10), Normal + IVA group (*n* = 10), CVMC + IVA group (*n* = 20), and CVMC2 group (*n* = 20). Mice in CVMC1 group, CVMC + IVA group, and CVMC2 group were inoculated intraperitoneally with CVB3 at the 1st, 14th, and 28th days using 0.20, 0.25, and 0.30 ml of 100TCID50 CVB3, respectively, and the mice in Normal control 1 group, Normal control 2 group, and Normal + IVA group received an equal volume of normal saline without CVB3 at the 1st, 14th, and 28th days. The day of virus inoculation was defined as day 1. Following day 42, groups 4 and 5 were gavaged with IVA (5 mg/kg-1⋅d-1) for 30 days until the 72nd day; concurrently, groups 3 and 6 received daily saline (5 mg/kg-1⋅d-1). IVA was dissolved in normal saline solution. The dose was selected according to previous experiments (Yue-Chun et al., 2012, 2016; Li et al., 2013). Mice in Normal control 1 group and CVMC1 group were killed for hematoxylin-eosin (HE) staining immediately following electrocardiographic (ECG) and echocardiographic analysis at the 42nd day. Mice in Normal control 2 group, Normal + IVA group, CVMC + IVA group, and CVMC2 group were killed for HE staining immediately following ECG and echocardiographic analysis at the 72nd day.

### Heart Rate and Echocardiographic Examination

The mice in Normal control 2 group, Normal + IVA group, CVMC + IVA group, and CVMC2 group were examined by ECG to exclude those with no-sinus rhythm after anesthetization with 5% chloral hydrate (0.01 ml/g) at the 72nd day. Transthoracic echocardiography was performed using a Sonos 5500 ultrasound machine (Phillips, United States) with a 12-MHz phased array transducer and real-time digital acquisition, storage, and review capabilities. The transducer was covered with a surgical latex glove finger filled with an ultrasound transmission gel to provide a standoff of 0.5–0.7 cm. The mice in each group were anesthetized intraperitoneally with 5% chloral hydrate (0.01 ml/g) at the 72nd day, as described previously (Li et al., 2010; Li-Sha et al., 2015). Two-dimensional, M-mode, Doppler flow images were obtained in the parasternal long-axis view after the chest was shaved. The left ventricular end-diastolic and end-systolic internal diameters (LVEDd, LVESd) were measured over the course of at least 3 consecutive cardiac cycles. The LVEF and fractional shortening (LVFS) were then simultaneously calculated.

### Pathologic Examination of HE-Stained Sections

After fixation with 10% neutral buffered formalin, the hearts were dehydrated and embedded in paraffin. Five-micrometer-thick sections were cut and mounted from warm water (40°C) onto adhesive microscope slides, deparaffinized, rehydrated, and stained with HE for histopathology assessment.

### Detection of Apoptosis

Apoptosis was detected in the myocardial tissue sections by using the terminal transferase-mediated DNA nick end labeling (TUNEL) assay. Apoptotic cells were identified using an *in situ* cell death detection kit (Roche, Switzerland). The myocardial tissue sections were incubated with 50 μl TUNEL reaction mixture containing terminal deoxynucleotidyl transferase (TdT) for 60 min at 37°C. The nuclei were then stained by DAPI. The positive apoptotic cells were labeled green and all the nuclei were labeled blue under the fluorescence microscope.

### Western Blot Analysis for Bax, Bcl-2, and Caspase-3

The expression of Bax, Bcl-2, and Caspase-3 protein in the myocardial tissue was determined by western blot analysis. Tissues were washed and lysed with a protein extraction reagent (Beyotime Biotechnology Company, Beijing, China). Equal amounts of protein (60 μg) were loaded and resolved on 10% SDS-polyacrylamide gels. The gels were transferred onto polyvinylidene difluoride (PVDF) membranes (Invitrogen Company, United States). The membranes were then blocked with TBS containing 5% (w/v) non-fat milk for 2 h at room temperature and sequentially incubated with either Bax, Bcl-2, or Caspase-3 rabbit anti-mouse antibody (1:100 dilution, Cell Signaling Technology, United States) or GAPDH antibody (1:1000 dilution, Cell Signaling Technology) for 2 h at room temperature. The membranes were then washed three times with tris-buffered saline containing Tween (TBST), followed by incubation with goat anti-rabbit HRP-conjugated antibody (1:10000 dilution, Beijing Zhongshan, China) for 2 h at room temperature. The membranes were then washed three more times with TBST and developed using hyperfilm-enhanced chemiluminescence (Amersham Biosciences, Freiburg, Germany). The resulting films from the membranes were subjected to semiquantitative analysis by AlphalmagerTM2200 gelatum imaging analysis system (Alpha Innotech Corporation, San Leandro, CA, United States).

### Statistical Analysis

All values are expressed as mean ± standard error (SE). Statistical analysis was performed by one-way analysis of variance (ANOVA), followed by Fisher’s protected least significant difference test. A value of *P* < 0.05 was considered significant.

## Results

### Physical Manifestations, Heart Rate and Echocardiography

The mice appeared ill 3 days after CVB3 inoculation. The untreated infected mice showed listlessness, lassitude, pilo-erection and poor appetite. After treatment with IVA, the physical activity and appetite of the infected mice gradually became better. The mice in Normal control 1 group, Normal control 2 group and Normal+IVA group showed no illness.

**FIGURE 1:**
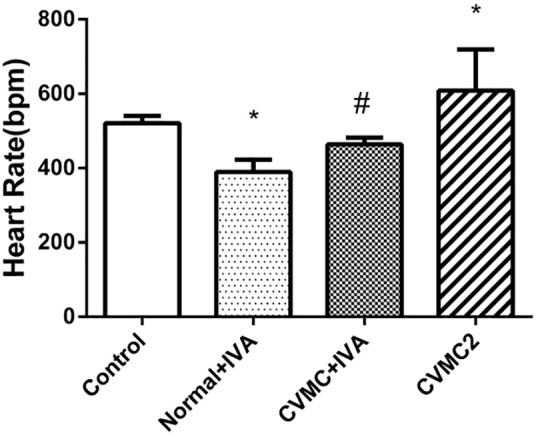
The heart rate in Normal control 2 group (*n* = 8), Normal + IVA group (*n* = 8), CVMC + IVA group (*n* = 8), and CVMC2 group (*n* = 8) on the 72nd day. Treatment with ivabradine (IVA) significantly reduced heart rate in CVMC+IVA group versus CVMC2 group. ^∗^*P* < 0.05 vs. Control (Normal control 2 group), ^#^*P* < 0.05 vs. CVMC2.

Compared with Normal control 2 group, the heart rate was significantly reduced (*P* < 0.05) in Normal+IVA group and increased (*P* < 0.05) in CVMC2 group. After treatment with IVA continuously for 30 days, the heart rate was significantly reduced (*P* < 0.05) in CVMC+IVA group versus CVMC2 group (**Figure 1**).

**FIGURE 2:**
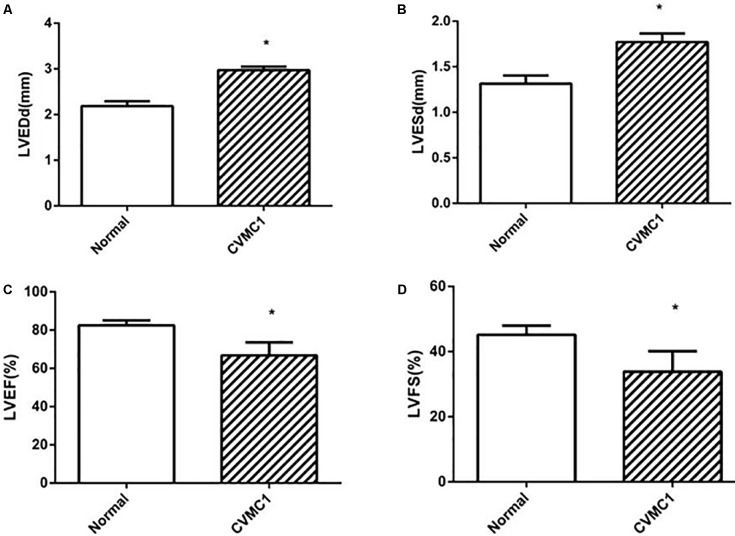
The echocardiography results in Normal control 1 group (*n* = 8) and CVMC1 group (*n* = 8) on the 42nd day. The LVEDd and LVESd were significantly dilated and the LVEF and LVFS in CVMC1 group were significantly lower than those of normal control group on day 42. **(A)** LVEDd = left ventricular end diastolic dimension, **(B)** LVESd = left ventricular end systolic dimension, **(C)** LVEF = left ventricular ejection fraction, and **(D)** LVFS = left ventricular shortening fraction. ^∗^*P* < 0.05 vs. Normal (Normal control 1 group).

The echocardiogram of CVMC1 group showed that the left ventricular internal diameter was dilated and the LVEF and LVFS were significantly lower than those of Normal control 1 group at the 42nd day (**Figure 2**).

**FIGURE 3:**
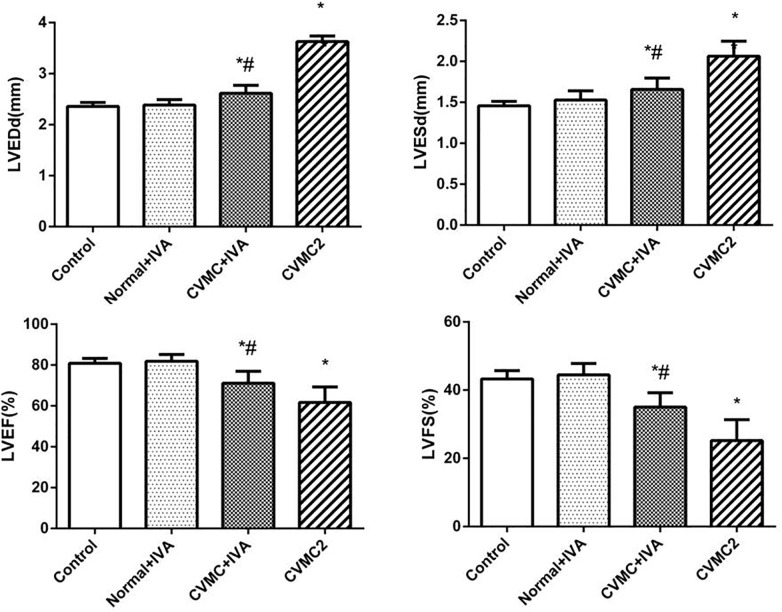
The echocardiography results in Normal control 2 group (*n* = 8), Normal + IVA group (*n* = 8), CVMC + IVA group (*n* = 8), and CVMC2 group (*n* = 8) on the 72nd day. Treatment with ivabradine (IVA) significantly reduced the left ventricular internal diameter and apparently increased the LVEF and LVFS in CVMC+IVA group as compared to that in CVMC2 group. LVEDd = left ventricular end diastolic dimension, LVESd = left ventricular end systolic dimension, LVEF = left ventricular ejection fraction, LVFS = left ventricular shortening fraction. ^∗^*P* < 0.05 vs. Control (Normal control 2 group), ^#^*P* < 0.05 vs. CVMC2.

At the 72nd day, IVA significantly reduced the left ventricular internal diameter and apparently increased the LVEF and LVFS in CVMC+IVA group as compared to that in CVMC2 group. No difference was observed between Normal+IVA group and Normal control 2 group (**Figure 3**).

### Pathologic Examination of HE-Stained Sections

**FIGURE 4:**
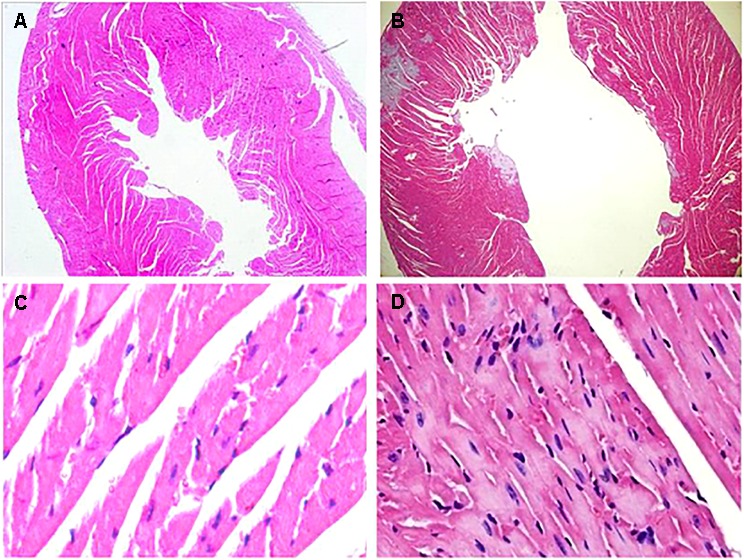
HE staining on the 42nd day. HE staining revealed that the heart was dilated and a large number of interstitial fibroblasts had infiltrated into the myocardium in CVMC1 group. **A** (× 40) and **C** (× 400) are for Normal control 1 group. **B** (× 40) and **D** (× 400) are for CVMC1 group.

At the 42nd day, HE staining revealed that the heart was dilated and a large number of interstitial fibroblasts had infiltrated into the myocardium in CVMC1 group (**Figure 4**).

**FIGURE 5:**
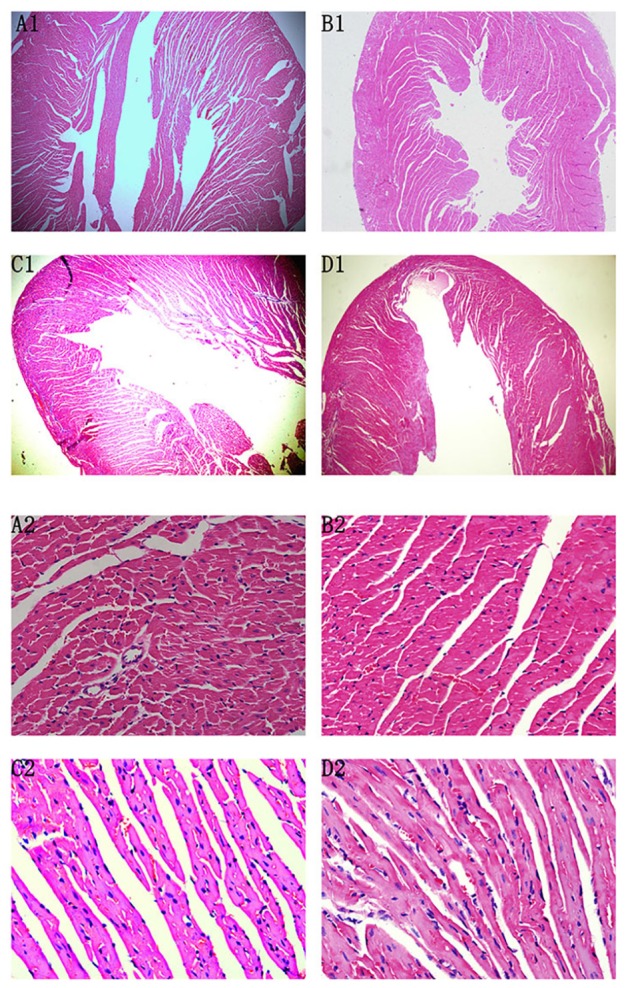
HE staining on the 72nd day. The heart chambers were shrunken and less interstitial fibroblasts infiltrated after treatment with ivabradine (IVA) in CVMC+IVA group as compared to that in CVMC2 group. **(A)** Normal control 2 group, **(B)** Normal+IVA group, **(C)** CVMC+IVA group, and **(D)** CVMC2 group.

At the 72nd day, HE staining revealed no differences between Normal control 2 group and Normal+IVA group. Compared to Normal control 2 group, the heart chambers were dilated and a large number of interstitial fibroblasts had infiltrated into the myocardium in CVMC2 group. Importantly, the heart chambers were shrunken and there was less infiltration of interstitial fibroblasts after treatment with IVA in CVMC+IVA group as compared to that in CVMC2 group, but more dilation and multiple fibroblasts were observed than those in Normal control 2 group (**Figure 5**).

### Cardiomyocyte Apoptosis

**FIGURE 6:**
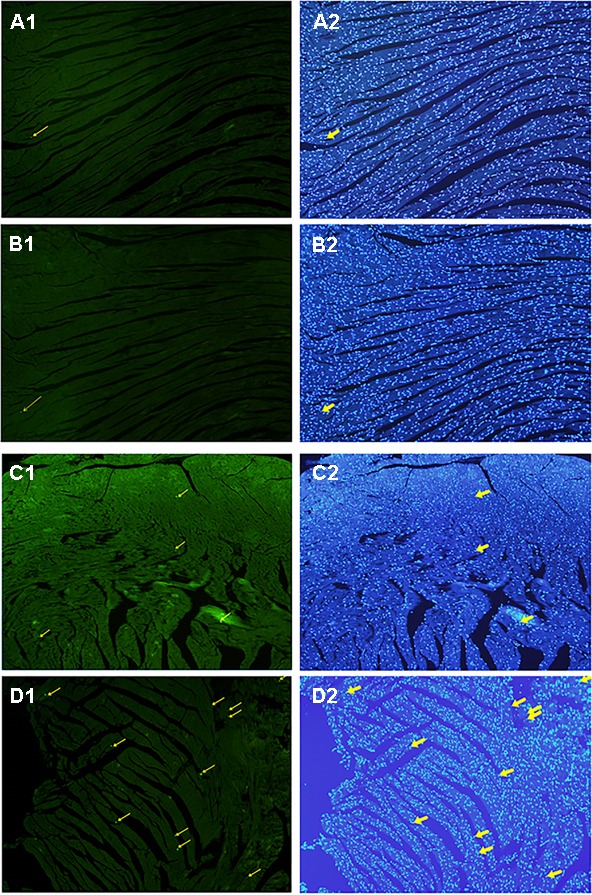
Detection of apoptotic cardiomyocytes with the TUNEL assay on the 72nd day. Apoptotic cells were labeled green and are indicated by the yellow arrows (left panel) and the corresponding cardiomyocyte were stained blue with DAPI (right panel). **(A)** Normal control 2 group, **(B)** Normal+IVA group, **(C)** CVMC+IVA group, and **(D)** CVMC2 group.

**FIGURE 7:**
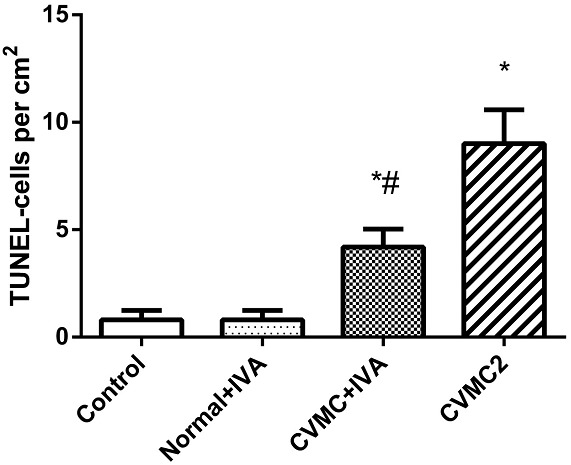
The positive apoptotic cells per cm^2^ of TUNEL assay in each groups (*n* = 8). The apoptotic percentages in CVMC+IVA group significantly attenuated after treatment with ivabradine (IVA) compared with CVMC2 group. ^∗^*P* < 0.05 vs. Control (Normal control 2 group), ^#^*P* < 0.05 vs. CVMC2.

All myocardial nuclei were stained blue with DAPI. The positive apoptotic cell nuclei appeared condensed and rounded, showing typical morphological features of apoptotic cells (**Figure 6**). The TUNEL-positive cells in the myocardium of the CVMC mice in CVMC2 group were significantly increased compared to those in the normal mice in Normal control 2 group. The apoptotic percentages in CVMC+IVA group significantly attenuated after treatment with IVA for 1 month compared with CVMC2 group, but it was still apparently more than that of the normal mice in Normal control 2 group. The statistical analysis of positive apoptotic cells per cm^2^ is shown in **Figure 7**.

### Western Blot Analysis for Bax, Bcl-2, and Caspase-3

**FIGURE 8:**
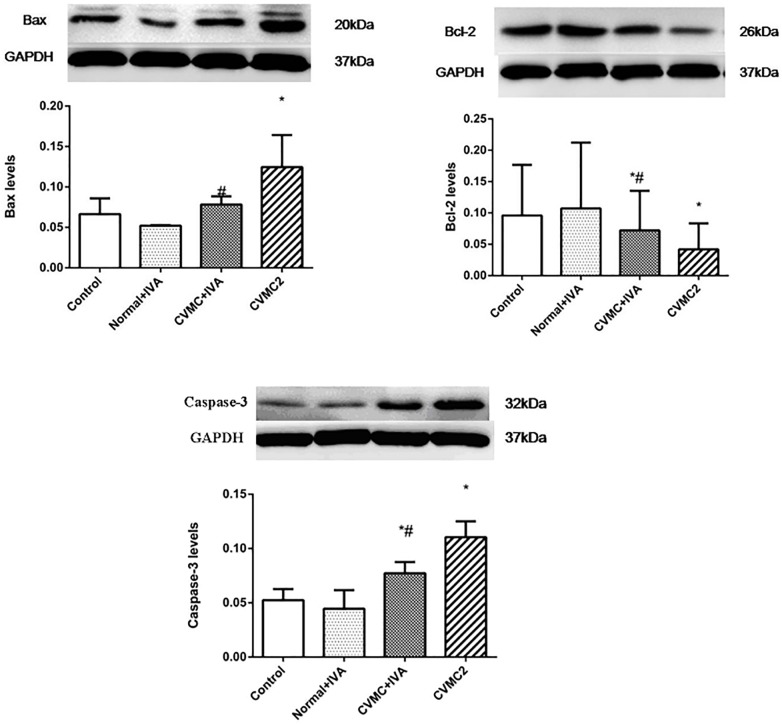
WB analysis for Bax, Bcl-2, and Caspase-3 in each groups (*n* = 8). Treatment with ivabradine (IVA) significantly reduced the expression levels of Bax and Caspase-3, and increased the expression level of Bcl-2. ^∗^*P* < 0.05 vs. Control (Normal control 2 group), ^#^*P* < 0.05 vs. CVMC2.

The expression levels of Bax and Caspase-3 in CVMC2 group were significantly higher than those in Normal control 2 group and were apparently reduced in CVMC+IVA group after treatment with IVA versus CVMC2 group. Bcl-2 showed high expression in Normal control 2 group and Normal+IVA group and low expression in CVMC2 group, but its expression level in CVMC+IVA group was higher than that in CVMC2 group. None of these proteins showed any difference in the expression levels between Normal control 2 group and Normal+IVA group (**Figure 8**).

## Discussion

The present study demonstrates that IVA significantly reduced the heart rate of CVMC mice with amelioration of CVMC and lessened the impairment of left ventricular function. IVA can downregulate the Bax level and upregulate the Bcl-2 level in CVMC mice and then reduce the caspase-3 level to inhibit cardiomyocyte apoptosis.

In agreement with previous studies (Aretz et al., 1987), we found that repetitive coxsackievirus infection induces CVMC and cardiac dilatation. According to the Dallas criteria (Aretz et al., 1987), CVMC is characterized by the accumulation of the connective tissue in the heart muscle, i.e., fibrosis. HE staining of the heart tissue in CVMC1 group (**Figures 2A,C**) showed a more dilated left ventricle, pronounced stromal fibroblast hyperplasia, and loosely arranged muscle fiber as compared to that in Normal control 1 group ((**Figures 2B,D**) at the 42nd day. Therefore, we considered that the mice in CVMC group on the 42nd day were in the CVMC stage. The World Health Organization has defined DCM as diseases of myocardial dilatation and impaired contraction of the left ventricle or both ventricles, which might be caused by idiopathic, familial/genetic, viral, or immune factors (Richardson et al., 1996). As compared to normal mice, the mice in CVMC1 group showed an expansion in the left ventricular volume, with an apparent reduction in the LVEF and LVFS; on the basis of this finding, we assumed that the mice on the 42nd day had not progressed into DCM. Thus, we considered that the model of CVMC was successfully established according to the findings of HE staining and echocardiogram.

Kannel et al. (Kannel et al., 1987) revealed that a high resting heart rate could increase the overall mortality regardless of gender and age in 5070 healthy people with 30 years of follow-up. Another American epidemiological investigation showed that a higher resting heart rate increased the risk of cardiovascular events and sudden death, but was not correlated with gender, race, or age (Gillum et al., 1991). Hence, many scholars consider that a higher resting heart rate should be regarded as a risk factor along with hypertension, diabetes, hyperlipidemia, smoking, etc. (Mensink and Hoffmeister, 1997; Palatini and Julius, 2004; Jouven et al., 2005; Palatini, 2007; Bohm et al., 2010). Our previous study confirmed that IVA could significantly reduce the heart rate and mortality in mice with acute viral myocarditis (Yue-Chun et al., 2012; Li et al., 2013). Therefore, appropriately and gradually reducing the heart rate is gaining attention as a new strategy for the prevention and treatment of cardiovascular diseases. Although beta-blockers can slow down the heart rate, their use is limited because of their negative inotropic and negative blood pressure effects (Cheng et al., 2016). IVA has been used in patients who could not tolerate beta blockers simply because of its ability to slow down the heart rate without any effect on contractility and blood pressure. The SHIFT trial revealed that IVA could obviously decrease cardiovascular events by reducing heart rate in heart failure patients (Bohm et al., 2010; Tardif et al., 2011). Riesen et al. (Riesen et al., 2012) found that IVA could not only significantly reduce the heart rate in cats with cardiomyopathy but also in normal cats. In the present study, we found that IVA could apparently decrease the heart rate of normal mice, but did not have any effects on left ventricular internal diameter, LVEF, and LVFS. Thus, we concluded that IVA simply decreased the sinus heart rate and did not have negative inotropic effects similar to those by beta-blockers. Many research studies have confirmed that IVA could improve cardiovascular outcomes and reverse ventricular remodeling. Ceconi et al. (Ceconi et al., 2011) considered that IVA could prevent or even reverse remodeling of the post-infarction rat heart in the chronic phase, and not in the acute phase, because it most likely required time to exert its full beneficial anti-remodeling effects and the more apparent heart function improvements occurred after longer treatment with IVA. IVA treatment significantly attenuated myocardial lesions and fibrosis, and decreased the expression of myocardial collagen I and collagen III in an experimental murine myocarditis model in our previous study (Yue-Chun et al., 2016). In this study, we found that the heart rate of untreated mice in CVMC2 group was faster than that of the normal mice, and the LVEF and LVFS were significantly reduced, but IVA treatment reduced the heart rate, shrunk the left ventricular chambers, and apparently improved LVEF and LVFS. From these results, we concluded that long-term treatment with IVA could improve cardiac diastolic left ventricular blood filling by reducing the heart rate to increase coronary blood flow and oxygen supply and reduce oxygen consumption and then cardiac function. After treatment with IVA for 30 days, the left ventricular internal diameter in CVMC + IVA group apparently decreased compared to that of the untreated CVMC mice in CVMC2 group; therefore, we concluded that IVA exerted protective effects on the CVMC mice and could prevent or reverse ventricular remodeling.

Huber et al. (Huber et al., 1999) confirmed abundant cardiomyocyte apoptosis in CVMC mice induced by CVB3. Our previous study indicated that cardiomyocyte apoptosis occurred in acute viral myocarditis, and the apoptotic cells were apparently reduced after treatment with carvedilol for 14 days, but only slightly lessened with IVA (Yue-Chun et al., 2012). Chen et al. (2014) found that IVA could improve cardiac output of diabetic mice by reducing cardiomyocyte apoptosis. Becher et al. (Becher et al., 2012) also concluded that IVA could improve left ventricular function in heart failure mice by decreasing cardiomyocyte apoptosis. Yamada et al. (Yamada et al., 1999) demonstrated that a certain amount of apoptotic myocardial cells existed in the CVB3-induced congestive heart failure model, and the left ventricular function was progressively degenerated following abundant loss of ventricular muscle cells resulting from cardiomyocyte apoptosis. Our results confirmed that there had been a much higher number of apoptotic myocardial cells in CVB3-induced CVMC mice (**Figure 6D**), and the apoptotic cells were significantly reduced after treatment with IVA for 30 days in CVMC+IVA group compared to that in the CVMC mice (**Figure 7**). Thus, we think that IVA has an anti-apoptotic effect. The apoptotic myocardial cells slightly reduced in acute viral myocarditis treated with IVA for only 14 days (Yue-Chun et al., 2012), but significantly decreased in CVMC treated with IVA for 30 days, although they were still markedly increased when compared to those of normal mice. In conclusion, we consider that the anti-apoptotic effects of IVA require a long time to become apparent, in addition to its more obvious anti-apoptotic effects in long-term treatment.

Mitochondria have increasingly become recognized as important organelles in regulating the apoptosis of cells, and cytochrome *c* (Cyt C) released from the mitochondria is considered the key component in the mitochondrial pathway of apoptosis (Vander Heiden and Thompson, 1999; Venteo et al., 2010). Many researchers have confirmed that the mitochondrial pathway is regulated by the balance between pro-apoptosis and anti-apoptosis proteins of Bcl-2 family members (Adams and Cory, 1998; Colston et al., 1998; Vander Heiden and Thompson, 1999; Antonsson et al., 2001; Carthy et al., 2003; Venteo et al., 2010). The expression levels of Bax were positively correlated with cardiomyocyte apoptosis in myocarditis, but the opposite was true for Bcl-2 (Joo et al., 2003). If Bax is highly expressed, it forms a homologous dimmer, which leads to cell apoptosis. If Bcl-2 is highly expressed, it forms a homologous dimmer, which prevents cell apoptosis. However, if Bax and Bcl-2 form a heterodimer, Bax will suppress the anti-apoptotic effect of Bcl-2, and thus, the proportion of the two proteins determines the apoptosis or survival of cells (Adams and Cory, 1998; Colston et al., 1998; Antonsson et al., 2001; Carthy et al., 2003). When it is triggered by extracellular signals, Bax acts on the mitochondrion, increasing the mitochondrial membrane permeability, which releases Cyt C and other proteins. Cyt C, apoptosis protease activating factor (Apaf-1), and Caspase-9 can form an apoptosome to autocatalyse caspase-9, thereby leading to the activation of downstream caspases such as Caspase-3 and Caspase-7 (Adams and Cory, 1998; Hu et al., 2014). Chen et al. (Hu et al., 2014) showed that cardiomyocyte apoptosis was present in diabetic mice, and IVA could decrease the expression of Bax, Caspase-3, and the apoptosis of myocardial cells, and significantly improve cardiac function. The high expression of Bax and Caspase-3, low expression of Bcl-2, and excessive cardiomyocyte apoptosis in CVMC mice indicated that the mitochondrial pathways are involved in CVMC myocardial apoptosis. The expression of Bax and Caspase-3 was reduced, the expression of Bcl-2 was increased, and the apoptotic cells were significantly decreased in CVMC mice treated with IVA; these results showed that IVA could attenuate the expression of Caspase-3 by downregulation of Bax and upregulation of Bcl-2 to prevent deterioration of cardiac function resulting from ventricular myocyte loss by cardiomyocyte apoptosis.

## Author Contributions

LY-C and CM designed the whole study. LY-C, GL-S, LL, ZD-P, SZ-W, GX, CG-Y, LJ, and LJ-F performed the experiments. GL-S and LL wrote the paper. All authors read and approved the final manuscript.

## Conflict of Interest Statement

The authors declare that the research was conducted in the absence of any commercial or financial relationships that could be construed as a potential conflict of interest. The reviewer TG and handling Editor declared their shared affiliation.
