# Corrigendum: Ivabradine Treatment Reduces Cardiomyocyte Apoptosis in a Murine Model of Chronic Viral Myocarditis

**DOI:** 10.3389/fphar.2019.01126

**Published:** 2019-09-27

**Authors:** Ge Li-Sha, Liu Li, Zhou De-Pu, Shi Zhe-Wei, Gu Xiaohong, Chen Guang-Yi, Li Jia, Lin Jia-Feng, Chu Maoping, Li Yue-Chun

**Affiliations:** ^1^Department of Pediatric Emergency, The Second Affiliated Hospital and Yuying Children’s Hospital of Wenzhou Medical University, Wenzhou, China; ^2^Department of Cardiology, The Second Affiliated Hospital and Yuying Children’s Hospital of Wenzhou Medical University, Wenzhou, China; ^3^Department of Cardiology, The First College of Clinical Medical Sciences, China Three Gorges University, Yichang, China; ^4^Children’s Heart Center and Department of Pediatrics, The Second Affiliated Hospital and Yuying Children’s Hospital of Wenzhou Medical University, Wenzhou, China

**Keywords:** coxsackievirus, chronic viral myocarditis, heart rate, ivabradine, cardiomyocyte apoptosis

In the original article, there was a mistake in **Figure 5A1 and A2** as published. The incorrect images were erroneously inserted. The corrected **Figure 5** appears below.

**Figure 5:**
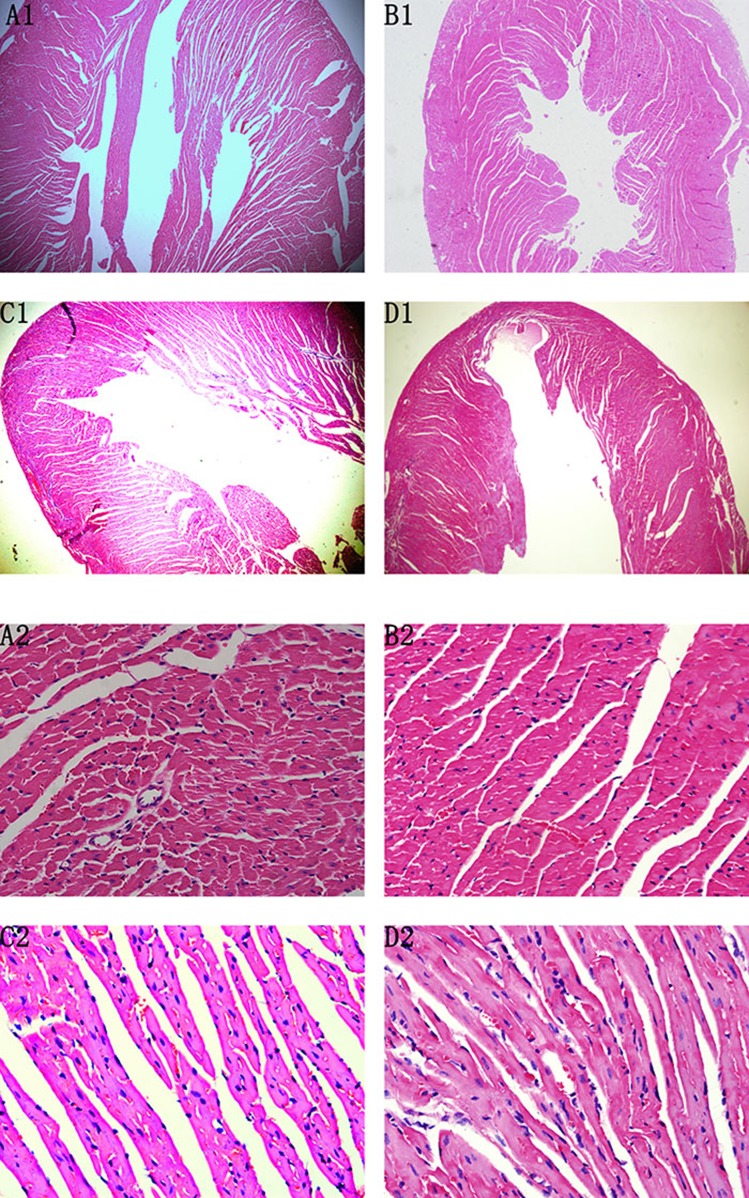
HE staining on the 72nd day. The heart chambers were shrunken and less interstitial fibroblasts infiltrated after treatment with ivabradine (IVA) in CVMC+IVA group as compared to that in CVMC2 group. **(A)** Normal control 2 group, **(B)** Normal+IVA group, **(C)** CVMC+IVA group, and **(D)** CVMC2 group.

The authors apologize for this error and state that this does not change the scientific conclusions of the article in any way. The original article has been updated.

